# Prevalence and influence of tuberculosis in the neurologic manifestations of the Human T cell lymphotropic virus type 1 (HTLV-1)

**DOI:** 10.1186/1742-4690-11-S1-P6

**Published:** 2014-01-07

**Authors:** Maria de Lourdes Bastos, Anselmo Souza, Natália Carvalho, Yuri Neves, Silvane Santos, Edgar M Carvalho

**Affiliations:** 1 Serviço de Imunologia, Complexo Hospitalar Universitário Professor Edgard Santos, Universidade Federal da Bahia, Salvador-BA, Brazil; 2 Escola Bahiana de Medicina e Saúde Pública, Salvador-BA, Brazil; 3 Hospital Especializado Octávio Mangabeira, Salvador-BA, Brazil; 4 Instituto Nacional de Ciência e Tecnologia de Doenças Tropicais (INCT-DT), Brazil

## 

HTLV-1 infects mainly T cells, leading to activation and cellular proliferation with exaggerated production of pro-inflammatory cytokines. The majority of HTLV-1 infected patients are considered as HTLV-1 carriers, but 5% will develop HTLV-1 associated myelopathy (HAM) and about 15% overactive bladder, an oligosymptomatic form of HAM. HTLV-1 is associated with increased susceptibility to *Mycobacterium tuberculosis* infection but up to now it is unknown if HTLV-1 influences the severity of tuberculosis and if tuberculosis may influence the outcome of HTLV-1. The aims of this study were to determine the prevalence and severity of tuberculosis (TB) in HTLV-1 infected patients and analyze whether TB influences the outcome of HTLV-1 infection. This is a cross-sectional study, in which the prevalence of tuberculosis was analyzed in 166 HTLV-1 infected individuals. Cytokine productions were determined by ELISA, the proviral load was evaluated by PCR. Tuberculosis occurred in 33 (22%) of cases and 70 (42%) had a positive tuberculin skin test (TST) (latent tuberculosis). The majority of the cases did not have severe tuberculosis. There was no difference in the proviral load, but there was a higher frequency of HAM/TSP in the group with tuberculosis than in HTLV-1 infected subjects without TB (P<0.05). This study shows that the prevalence of *M.tuberculosis* infection is 6 fold higher in HTLV-1 infected individuals than that described in the Brazilian population the majority of co-infected had latent tuberculosis and HTLV-1 may influence progression from infection to HAM/TSP.

## Funded by

National Institute of Health (NIH AI 079238) and CNPq.

